# Supplementary material for: MetaRibo-Seq measures translation in microbiomes
Source: Nat Commun. 2020 Jun 29;11:3268. doi: 10.1038/s41467-020-17081-z (PMC7324362; doi:10.1038/s41467-020-17081-z)
Supplement: Supplementary file 10 — Supplementary Data 7 [file 41467_2020_17081_MOESM10_ESM.zip › File2/Confidence_High_Taxonomy/335591_out.krona.html]

Javascript must be enabled to view this page.

members
magnitude
magnitudeUnassigned
count
unassigned
taxon
rank

335591\_out

8

2
superkingdom
8

8
phylum
1239

186801
class
8

186802
order
8


SRS015663\_contig\_number\_22399SRS016095\_contig\_number\_20872SRS023914\_contig\_number\_17052SRS050801\_contig\_number\_126SRS053356\_contig\_number\_28567SRS077392\_contig\_number\_25640SRS098571\_contig\_number\_42818
1898207
species
7

1
family
31979

1485
1
genus

1
species
1262815

SRS051031\_contig\_number\_23320
